# Effects of poor maternal diet during gestation are detected in F2 offspring

**DOI:** 10.1093/tas/txae055

**Published:** 2024-04-09

**Authors:** N M Tillquist, S A Reed, A S Reiter, M Y Kawaida, E C Lee, S A Zinn, K E Govoni

**Affiliations:** University of Connecticut, Department of Animal Science, Storrs, CT 06269, USA; University of Connecticut, Department of Animal Science, Storrs, CT 06269, USA; University of Connecticut, Department of Animal Science, Storrs, CT 06269, USA; University of Connecticut, Department of Animal Science, Storrs, CT 06269, USA; University of Connecticut, Department of Kinesiology, Storrs, CT 06269, USA; University of Connecticut, Department of Animal Science, Storrs, CT 06269, USA; University of Connecticut, Department of Animal Science, Storrs, CT 06269, USA

**Keywords:** growth, maternal nutrition, metabolism, multigenerational, sheep

## Abstract

Poor maternal nutrition of F0 ewes impairs F1 offspring growth, with minimal differences in glucose tolerance or select metabolic circulating factors, and independent of differences in residual feed intake (RFI). To determine if poor maternal nutrition in F0 ewes alters F2 offspring growth, circulating leptin, feed efficiency, or glucose tolerance, F0 ewes (*n* = 46) pregnant with twins were fed 100% (control), 60% (restricted), or 140% (over) of National Research Council requirements from days 30 ± 0.02 of gestation until parturition. At 16 to 19 mo of age, female F1 (*n* = 36) offspring were bred to generate F2 offspring [CON-F2 (*n* = 12 ewes; 6 rams), RES-F2 (*n* = 7 ewes; 13 rams), or OVER-F2 (*n* = 13 ewes; 9 rams) corresponding to diets of the granddam (F0)]. Lamb body weights (BW) and blood samples were collected weekly from days 0 to 28 and every 14 d until day 252 of age. Circulating leptin was measured in serum at days 0, 7, 14, 56, 210, and 252. An intravenous glucose tolerance test was performed at days 133 ± 0.28. At days 167 ± 0.33, individual daily intake was recorded over a 77-d feeding period to determine RFI. Rams were euthanized at days 285 ± 0.93, and body morphometrics, loin eye area (LEA), back fat thickness, and organ weights were collected and bone mineral density (BMD) and length were determined in the right hind leg. During gestation, OVER-F1 ewes tended to be 8.6% smaller than CON-F1 ewes (*P* ≤ 0.06). F2 offspring were of similar BW from birth to day 70 (*P* ≥ 0.20). However, from days 84 to 252, RES-F2 offspring tended to be 7.3% smaller than CON-F2 (*P* ≤ 0.10). Granddam diet did not influence F2 ram body morphometrics, organ or muscle weights, LEA, adipose deposition, or leg BMD (*P* ≥ 0.84). RES-F2 (−0.20) and CON-F2 (−0.45) rams tended to be more feed efficient than CON-F2 ewes (0.31; *P* ≤ 0.08). No effects of granddam diet were observed on glucose or insulin average or baseline concentrations, area under the curve, first-phase response, or ratio (*P* ≥ 0.52). However, CON-F2 rams (297 mg/dL ± 16.5) had a greater glucose peak compared with RES-F2 rams (239 mg/dL ± 11.2; *P* = 0.05). Peak insulin concentrations were not influenced by granddam diet (*P* = 0.75). At d 56, RES-F2 and OVER-F2 offspring had 53.5% and 61.8% less leptin compared with CON-F2 offspring, respectively (*P *≤ 0.02). These data indicate that poor maternal nutrition impacts offspring growth into the second generation with minimal impacts on offspring RFI, glucose tolerance, and circulating leptin.

## Teaser

Poor maternal nutrition during gestation impacts the growth of second-generation offspring, despite adequate nutrition of first-generation offspring. These effects are independent of differences in residual feed intake and body morphometrics.

## Introduction

In the livestock industry, poor maternal diet can negatively influence offspring production efficiency. Poor maternal diet can result from lack of availability of nutritional resources (restriction) or from poor livestock management (over-feeding). There is evidence that negative attributes such as insulin resistance, altered body composition, and decreased body weight (BW) gain persist across generations despite offspring being managed similarly in their nutrition and environment. Shasa et al. (2015) demonstrated that maternal over-feeding in sheep during gestation can disrupt the early postnatal leptin surge in female F1 and F2 offspring as well as increase circulating glucose, insulin, and cortisol. Similarly, F2 ewes from over-fed granddams exhibit greater cortisol and instance of insulin resistance during gestation compared with F2 ewes from control-fed granddams (Pankey et al., 2021). Despite differences in metabolic status and BW gain in F2 ewes from over-fed granddams, F3 neonates were of similar BW, body fat percent, fat mass, and lean mass compared with controls (Pankey et al., 2021). Together, these data indicate that poor maternal diet can induce metabolic dysregulation in offspring over at least two generations using an over-fed livestock animal model.

Nutrient restriction can occur in livestock animals during times of drought or during times of increased nutrient demand such as gestation, where the dam is unable to eat enough to satisfy nutrient requirements. Nutrient restriction during gestation can decrease growth and alter metabolism in offspring (Hoffman et al., 2017; Long et al., 2021). Altered metabolic efficiency as a result of poor maternal diet in livestock can increase cost of production and decrease product quality. However, there are limited studies using a livestock model of nutrient restriction that investigate offspring into the second generation. Evidence from rodent models has provided us with an understanding of the consequences that exist due to nutrient restriction during gestation. Specifically, protein restriction during gestation in F0 rats impairs glucose tolerance of both F1 and F2 adult offspring (Zambrano et al., 2005; Pinheiro et al., 2008). Furthermore, impaired glucose tolerance has been reported in rats in the F3 generation (Benyshek et al., 2006), thus demonstrating that the effects of poor maternal nutrition are evident across multiple generations. Despite evidence of the consequences of maternal diet on offspring metabolism across multiple generations in rodent models, there are limited studies that investigate the multigenerational effects of poor maternal diet using livestock species. Understanding how both restricted- and over-feeding during gestation impact offspring growth and metabolism is warranted. Therefore, the objective of this experiment was to evaluate the effects of restricted- and over-feeding in the same model during gestation on grandoffspring (F2) growth, feed efficiency, and metabolism. We hypothesized that F2 offspring from restricted- and over-fed granddams would have reduced BW, feed efficiency, and glucose tolerance, altered circulating leptin, and greater adiposity than F2 offspring from control-fed granddams.

## Materials and Methods

### F1 Ewe Management

All procedures were completed in accordance with guidelines established and approved by the University of Connecticut Animal Care and Use Committee (A22-017).

**Figure 1.**
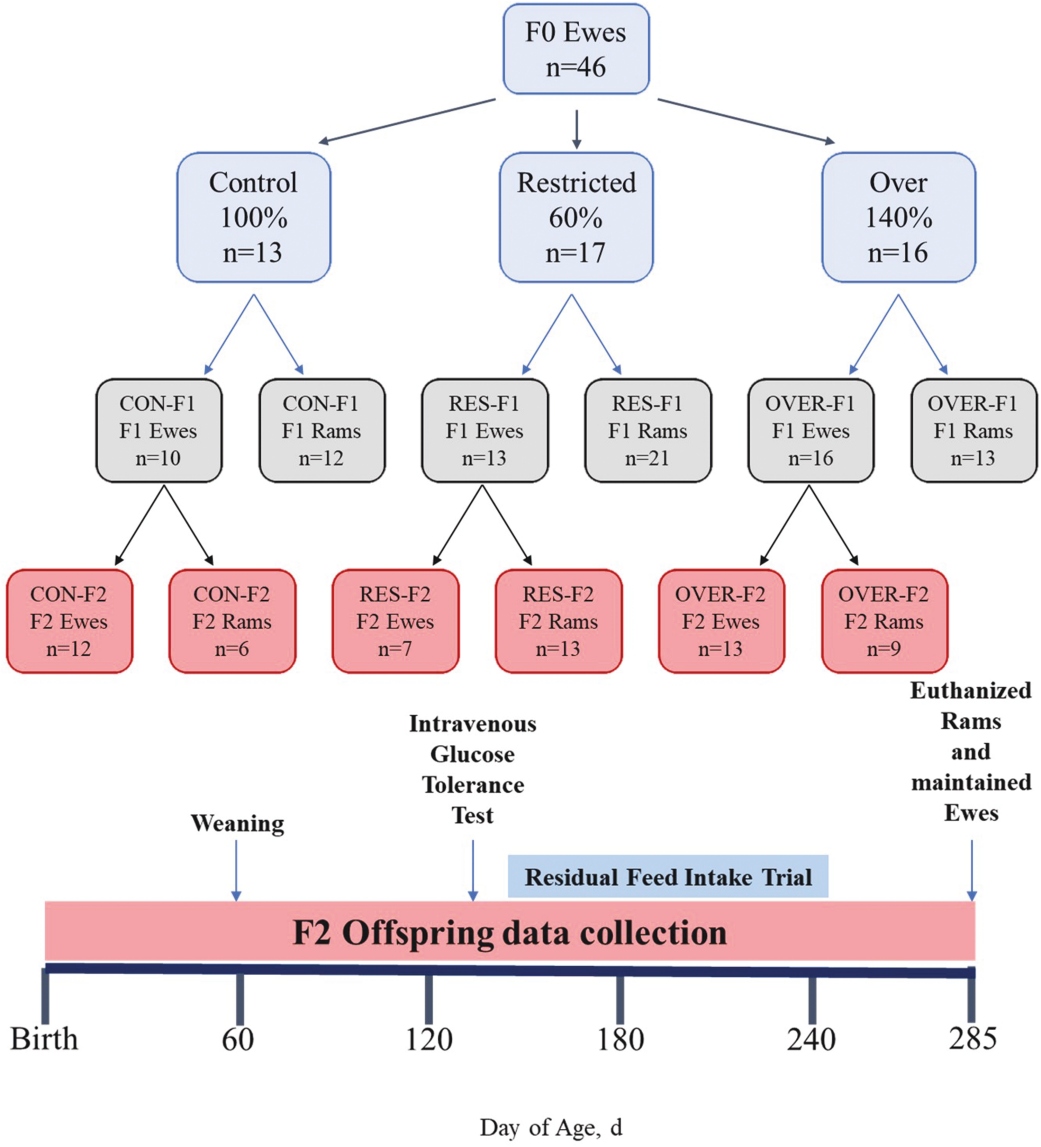
Experimental design. To evaluate the multigenerational effects of restricted- and over-feeding on postnatal grandoffspring (F2) development, pregnant ewes (F0; *n* = 46) were housed in individual pens on day 20 of gestation and transitioned to a control complete pelleted diet. At day 26 of gestation, F0 ewes were blocked by body weights and assigned to one of the three dietary treatments (control: 100%; restricted: 60%; or over: 140% of NRC requirement for TDN for ewes pregnant with twins). F1 ewes were fed a control diet and between 16 and 19 mo of age were bred with 1 of the 2 genetically similar Dorset rams to generate the F2 population. F2 offspring were weaned at day 60 and underwent intravenous glucose tolerance tests and residual feed intake trials at days 133 and 167 of age, respectively (Tillquist et al., 2023). F2 ram offspring were necropsied at day 285 of age and F2 ewes were maintained on a control diet for future breeding.

A detailed description of the experimental design, animals, and diets used was previously reported (Tillquist et al., 2023). To evaluate the multigenerational effects of poor maternal diet, first-generation (F1) ewes were used to generate an F2 population of offspring. F1 ewes were maintained on a 16% sheep grain (Table 1; Pleasant View Farms Inc., Somers, CT) and ad libitum second-cutting hay (Table 1). The F1 ewes (*n* = 37; Figure 1 (CON-F1, *n* = 10; RES-F1, *n* = 12; OVER-F1, *n* = 15]) were estrus synchronized between 16 and 19 mo of age using controlled intravaginal drug release devices (CIDRs; Zoetis, Parsippany-Troy Hills, NJ) and prostaglandin (Lutalyse, Zoetis, Parsippany-Troy Hills, NJ) and bred by live cover with one of two genetically related Dorset rams as previously described (Reed et al., 2014; Hoffman et al., 2016a; Pillai et al., 2017; Tillquist et al., 2023). Breeding rams were not genetically related to the breeding rams used to generate the F1 offspring. Day 0 of pregnancy was considered when a raddle mark was observed on the rump of the ewe. Pregnancy with twins was confirmed using transabdominal ultrasound (Jones et al., 2016). Three F1 ewes were pregnant with singletons (CON-F1, *n* = 1; RES-F1, *n* = 2). Four ewes did not get pregnant and were removed from the study (RES-F1, *n* = 1; OVER-F1, *n* = 3). One ewe died during late gestation for reasons unrelated to treatment (OVER-F1, *n* = 1; data not included in the figure or statistical analysis). Ewes were allowed to lamb naturally and remain with the lambs group housed until offspring were weaned.

**Table 1.** Chemical composition of F1 and F2 offspring diets

Nutrient analysis^1^	F1 Ewes			F2 offspring		
	Complete pellet^2^	Sheep grain	Second cutting hay	Creep feed^3^	Grower feed^4^	Complete pellet^5^
Moisture, %	9.45	12.00	16.10	10.80	13.50	9.78
Dry matter, %	90.57	88.00	83.90	89.20	86.60	90.23
Crude protein, %	18.48	21.60	15.80	21.20	18.50	17.78
Adjusted crude protein, %	18.48	21.60	15.80	21.20	18.50	17.78
ADF, %	25.95	4.00	30.70	11.70	11.70	27.30
aNDF^6^, %	40.28	7.50	54.50	18.80	24.70	37.48
TDN, %	74.33	85.00	58.00	81.00	79.00	75.25
DE, Mcal/kg	2.80	3.68	2.25	3.37	3.26	2.87
NEL, Mcal/kg	1.75	2.01	1.23	1.91	1.87	1.78
NEM, Mcal/kg	1.77	2.14	1.17	2.00	1.94	1.80
NEG, Mcal/kg	1.15	1.46	0.84	1.34	1.30	1.17
Calcium, %	1.42	1.42	0.38	0.75	1.45	1.60
Phosphorus, %	0.54	0.42	0.25	0.63	0.68	0.62
Magnesium, %	0.34	0.20	0.23	0.28	0.32	0.29
Potassium, %	2.16	1.12	2.32	1.58	1.00	1.76
Sodium, %	0.51	0.33	0.06	0.25	0.35	0.54
Iron, mg/kg	358.50	212.00	161.00	394.00	270.00	390.50
Zinc, mg/kg	103.17	70.00	22.00	316.00	187.00	125.5
Copper, mg/kg	11.67	8.00	8.00	6.00	4.00	16.75
Manganese, mg/kg	86.33	52.00	59.00	158.00	125.00	101.75
Molybdenum, mg/kg	5.83	3.10	0.80	12.50	0.50	6.48

^1^Nutrient analyses were performed by Dairy One, Inc. (Ithaca, NY).

^2^F1 offspring were fed Complete Feed from days 154 to 282 of age. Values are presented as an average analysis of four deliveries.

^3^Offspring were fed Creep Feed until day 120 of age.

^4^Offspring were fed Grower Feed from days 121 to 153 of age.

^5^F2 offspring were fed Complete Feed from days 154 to 282 of age. Values are presented as an average analysis of three deliveries.

^6^Neutral detergent fiber digested in amylase and sodium sulfite.

#### F1 Ewe sample collection.

Body weight and body condition score (BCS) of F1 ewes were recorded monthly during gestation. Body weight and BCS from each F1 ewe were recorded when rams were added into the pen, at time of marking, and monthly until day 120 of gestation.

### F2 Offspring Management

After birth, F2 lambs were maintained with their F1 dam and allowed ad libitum access to creep feed (Table 1; Home Fresh 18 Sheep Starter, Blue Seal, Litchfield, CT) and second cutting hay until weaning at 60 d of age. F2 offspring (*n* = 60; Figure 1) are referred to as CON-F2 (*n* = 12 ewes; 6 rams), RES-F2 (*n* = 7 ewes; 13 rams), or OVER-F2 (*n* = 13 ewes; 9 rams), corresponding to the diets of the granddam (F0; Tillquist et al., 2023). Two F2 offspring died at birth for reasons unrelated to the experiment (CON-F2, *n* = 1; OVER-F2, *n* = 1; not included in statistical analysis). One offspring (OVER-F2, *n* = 1) died at 142 d of age unrelated to the experiment. Data for this individual remained in the study for BW, and glucose and insulin analyses, but was removed from serum leptin analysis because post-weaning samples were not collected. After weaning (Figure 1), lambs were housed together and fed grower feed (Table 1; Home Fresh Shepherd 16, Blue Seal) to 100% of NRC requirements. An intravenous glucose tolerance test (IV-GTT) was performed on lambs at 133 ± 0.28 d of age, as previously described (Ford et al., 2007; Hoffman et al., 2014). At 120 ± 0.12 d of age, residual feed intake (RFI) was measured in F2 lambs utilizing a Super SmartFeed system (C-Lock Inc., Rapid City, SD) and complete pelleted feed (Table 1). Rams were euthanized at days 285 ± 0.93 of age, and ewes were maintained on complete pelleted feed to 100% of NRC requirements.

#### F2 offspring sample collection.

Following the same collection timeline used for the F1 offspring (Tillquist et al., 2023), F2 lamb BW and jugular blood samples (10 to 20 mL) were collected weekly for the first 4 wk and then every 14 d until 252 d of age. Crown-rump length (CRL), heart girth (HG), and hip height (HH) measurements were taken at days 0 and 120 of age. Lamb BCS was recorded at day 120 of age for all offspring and at day 285 of age for ram offspring. The F2 ram lambs were euthanized at days 285 ± 0.93 of age (Figure 1) by intravenous injection of Euthasol (Virbac, Fort Worth, TX) containing 390 mg/mL sodium pentobarbital and 50 mg/mL sodium phenytoin based on BW of the ram on the day of euthanasia (0.22 mL/kg BW). Immediately before euthanasia, CRL, HG, HH, scrotal circumference, and BCS were measured, and a final blood sample (20 mL) was collected. After euthanasia, loin eye area (cm^2^) and backfat thickness were measured. Longissimus (LM), semitendinosus (STN), and triceps brachii muscles, heart, liver, pancreas, adrenal glands, kidneys, and testes were removed and weighed. The right hind leg of each ram was removed for length and bone density analyses. Density was determined using a dual-energy x-ray absorptiometry (DXA; Lunar Prodigy; GE Healthcare, Madison, WI) scanner which generated bone mineral density values (g/cm^2^) for each individual leg.

### Intravenous Glucose Tolerance Test

A fasting IV-GTT was performed at days 133 ± 0.28 of age. The necks of lambs were shaved and cleaned with chlorhexidine (Durvet, Beaver Dam, WI) followed by 70% ethanol (Fisher Bioreagents, Pittsburgh, PA). A cannula (18 g × 2.5 in; Exel International, Quebec, Canada) was inserted into a jugular vein of each lamb 1 h before GTT to allow lambs time to recover. A single bolus injection of glucose (0.25 g/kg BW of a 50% dextrose solution; VetOne, Boise, ID) was infused via the jugular cannula. Blood samples (3 mL) were collected via the cannula at −30, −15, 0, 2, 5, 10, 15, 30, 60, and 120 min relative to glucose infusion, placed into heparin tubes (Greiner Bio-one), and stored on ice. Blood was centrifuged (3,000 × g for 30 min at 4 °C), and plasma was stored at −20 °C for insulin and glucose analyses.

### Residual Feed Intake

At 120 ± 0.12 d of age, a radio frequency identification ear tag (Allflex, Rathway, NJ) was placed in the right ear of each lamb following the manufacturer placement guidelines. At days 167 ± 0.33 of age, feed intake from each individual animal was measured for a 77-d feeding period to determine RFI (Koch et al., 1963; Arthur et al., 2001; Herd and Arthur, 2009; Cockrum et al., 2013) in which the animals had ad libitum access to a complete pelleted feed (F2 complete feed; Table 1). BW on two consecutive days were recorded at the beginning, mid-point, and end of the RFI trial. Average daily feed intake was calculated as total feed/days on feed. Feed conversion efficiency was calculated as total BW gained/feed consumed during the feeding period. Predicted feed intake was calculated by regressing the actual (measured) feed intake on metabolic mid-weight [MMWT; (Mid-BW)^0.75^] and average daily gain [final BW—starting (BW)/days on feed]. RFI was calculated by subtracting the actual feed intake from the predicted feed intake value that was calculated using the regression equation as previously described (Arthur et al., 2001). A negative RFI coefficient indicates that the animal consumed less than the predicted amount and is therefore considered more feed efficient.

### Glucose, Insulin, and Leptin Analysis

Plasma samples from the IV-GTT were analyzed for insulin and glucose at all collected time points. Plasma insulin concentrations were determined by an ovine insulin enzyme-linked immunoassay (ELISA; Mercodia Inc., Uppsala, Sweden) as previously described (Vaughan et al., 2016). The limit of detection for the insulin ELISA was 0.025 ng/mL, and the intra- and inter-assay coefficients were 4.69% and 3.81%, respectively. Cubic spline analysis was performed using an online data analysis tool (MyAssays Ltd.) for determination of insulin concentrations. Plasma glucose concentrations were determined using a colorimetric assay kit (Cayman Chemical, Ann Arbor, MI) as previously described (Hoffman et al., 2016a). The limit of detection for the glucose colorimetric assay was 0.23 mg/dL, and the intra-assay coefficient was 9.50%. For glucose analysis, plasma was diluted 1:10 for −30, −15, 0, 5, 10, 15, 30, 60, 120 min samples and 1:15 for 2 min sample. Serum samples collected from F2 offspring at days 0, 7, 14, 56, 210, and 252 of age were used for leptin analysis using a multi-species radioimmunoassay (RIA; MilliporeSigma, Burlington, MA). Samples are classified as preweaning (0, 7, 14, and 56) and mature (210, 252) timepoints. The limit of detection for the leptin RIA was 0.801 ng/mL, and the intra- and inter-assay coefficients were 1.83% and 4.13%, respectively. These kits have been successfully optimized for use with ovine samples (Hoffman et al., 2014; Soranno et al., 2021; Tillquist et al., 2023), and manufacturer instructions were followed.

### Statistical Analysis

Data were analyzed using the R programming language in the R Studio (version 4.2.2; R Core Team, 2021) on “Spotted Wakerobin” release for Windows, using the packages car (Fox and Weisberg, 2020), emmeans (Lenth et al., 2022), ggpubr (Kassambara, 2020), lme4 (Bates et al., 2015), nlme (Pinheiro et al., 2022), rstatix (Kassambara, 2022), and tidyverse (Wickham et al., 2019). Body weight, body morphometric, and circulating factors data were analyzed using a two-way or three-way mixed effects analysis of variance (ANOVA) to account for repeated measures with animal (random), maternal treatment (fixed), sex (fixed), and time/day (continuous) included in the model, where appropriate. Predicted feed intake was obtained through regression analysis of ADG and MMWT on actual daily feed intake. Residual value between actual and predicted intake was used as the RFI coefficient as previously described (Herd and Arthur, 2009; Cockrum et al., 2013). Baseline concentrations, area under the curve (AUC), first-phase response, and insulin-to-glucose ratio were determined as previously described (Hoffman et al., 2016b). Organ weights are expressed as g/kg BW to account for differences in offspring BW. Bone lengths were determined using ImageJ (version 1.53) and analyzed as a one-way ANOVA with maternal treatment as the fixed effect. Where appropriate, post hoc pairwise comparisons were made using emmeans. Statistical significance was considered at *P* ≤ 0.05 and a tendency at 0.05 < *P* ≤ 0.10.

## Results

### F1 Ewes

When F1 ewes moved into breeding pen (between 16 and 19 mo of age), CON-F1 ewes weighed 11.5% and 8.7% more than RES-F1 and OVER-F1 ewes, respectively (*P* ≤ 0.04; Table 2). On average, from day 30 of gestation to day 120, OVER-F1 ewes tended to weigh 8.6% less than CON-F1 ewes (*P* ≤ 0.06), and RES-F1 ewes did not differ (*P* ≥ 0.22). An interaction of treatment by time was not observed for F1 ewe BCS (*P* = 0.20); however, a main effect of treatment was detected where ewes from restricted-fed dams had greater (3.18 ± 0.04; *P* = 0.03) BCS relative to ewes from over-fed dams (3.00 ± 0.05). Animals from control-fed dams did not differ (3.10 ± 0.05; *P* ≥ 0.31).

**Table 2.** Effects of maternal diet on F1 Ewe BW at the beginning of breeding season and from days 0 to 120 of gestation

	Treatment^1^				
Item^2^	CON-F1	RES-F1	OVER-F1	SEM^3^	*P-*Value^4^
Breeding^5^	86.26^a^	76.31^b b^	78.70^b^	3.66	0.04
Day 0^6^	84.13^b^	78.99 ^b^	77.41 ^b^	2.38	0.12
Day 30	87.03^x^	81.76^xy^	79.16^y^	2.41	0.06
Day 60	95.15^a^	91.29^ab^	85.59^b^	2.76	0.02
Day 90	101.63^x^	97.71^xy^	93.49^y^	2.50	0.06
Day 120	111.50^a^	107.00^ab^	103.10^b^	2.80	0.05

Means with different superscripts ^(a-b)^ within row represent differences among treatments (*P* ≤ 0.05).

Means with different superscripts ^(x-y)^ within row represent trend among treatments (0.05 < *P* ≤ 0.10).

^1^F0 Dorset ewes pregnant with twins were fed 100%, 60%, or 140% of NRC requirements from day 30 of gestation until parturition. Offspring (F1) are referred to as CON-F1, RES-F1, OVER-F1, respectively.

^2^Values are presented as BW, kg.

^3^Largest SEM across treatment for each timepoint.

^4^ *P*-value for main effect of treatment at each timepoint.

^5^Ewes were weighed, balanced by BW and treatment, and placed into a pen with 1 of the 2 genetically similar rams. Breeding = BW on the day the ewes were put in with breeding rams.

^7^Day 0 refers to the date that a heavy breeding crayon marking was observed on the rump of the ewe.

### F2 Offspring

#### Growth and body morphometrics.

An effect of maternal diet on F1 offspring length of gestation was not detected (*P* = 0.34; CON-F1: 148 d ± 0.9, RES-F1: 147 d ± 0.9, OVER-F1: 145 d ± 0.8). A two-way interaction of treatment and time was detected for F2 offspring BW (*P *< 0.0001). Although granddam diet did not have a statistically significant effect on F2 offspring BW from days 0 to 70 of age (*P* ≤ 0.20; Table 3). At day 84 of age, RES-F2 were 10.9% lighter than CON-F2 offspring (*P *= 0.05), and OVER-F2 offspring tended to be 9.3% lighter than CON-F2 offspring (*P* = 0.09). At d 98 of age, RES-F2 offspring were 11.3% lighter (*P *= 0.03), and OVER-F2 offspring were 10.4% lighter compared with CON-F2 offspring (*P *= 0.03). On average, from days 112 to 154 of age, RES-F2 offspring tended to be 8.7% lighter than CON-F2 offspring (*P* ≤ 0.08) and OVER-F2 offspring did not differ (*P* ≥ 0.19). At d 196 of age, OVER-F2 offspring were 5.9% heavier than RES-F2 offspring (*P* = 0.05), and CON-F2 offspring did not differ (*P* ≥ 0.24). From days 224 to 252, RES-F2 offspring were 6.5% and 6.3% lighter than CON-F2 and OVER-F2 offspring, respectively (*P* ≤ 0.01). Averaged across the sampling periods, F2 ram lambs were 8.36 kg heavier than F2 ewe lambs (*P* < 0.0001).

**Table 3.** Effects of granddam diet on F2 offspring BW

	Treatment^1^				
Item^4^	CON-F2	RES-F2	OVER-F2	SEM^2^	*P*-value^3^
BW, kg					
Day 0	5.32^qq^	5.13^q^	5.21^qq^	0.23	1.00
Day 7	8.19 ^qq^	7.51^q^	7.31 ^qq^	0.31	0.84
Day 14	10.41 ^qq^	9.65^q^	9.14 ^qq^	0.37	0.71
Day 21	12.88 ^qq^	12.09^q^	11.50 ^qq^	0.45	0.67
Day 28	15.25 ^qq^	14.16^q^	13.77 ^qq^	0.62	0.63
Day 42	21.91 ^qq^	20.18^q^	19.49 ^qq^	0.98	0.29
Day 56	28.92 ^qq^	25.48^q^	26.15 ^qq^	1.31	0.20
Day 70	31.75 ^qq^	28.80^q^	29.91 ^qq^	1.46	0.49
Day 84	36.06^aq^	32.11^b^	32.69^ab^	1.49	0.05
Day 98	40.62^aq^	36.03^b^	36.41^bq^	1.65	0.03
Day 112	44.88^aq^	40.18^b^	42.05^ab^	1.75	0.02
Day 126	48.05^aq^	43.89^b^	46.00^ab^	1.73	0.04
Day 140	51.21^xq^	47.54^y^	48.38^xy^	1.76	0.08
Day 154	54.54^aq^	49.66^b^	52.19^ab^	1.84	0.01
Day 168	55.89^qq^	52.88^q^	54.49^qq^	2.19	0.17
Day 182	59.09^qq^	55.92^q^	59.13^q q^	2.07	0.15
Day 196	64.72^ab^	62.05^a^	65.76^bq^	1.28	0.05
Day 210	71.07 ^q^	67.74^q^	70.97^qq^	2.32	0.11
Day 224	74.15^aq^	70.39^b^	75.57^aq^	2.58	0.01
Day 238	78.02^aq^	71.94^b^	77.62^aq^	2.57	0.01
Day 252	79.03^aq^	73.90^b^	77.55^aq^	2.41	0.01

Means with different superscripts ^(a-b)^ within row represent differences among treatments (*P* ≤ 0.05).

Means with different superscripts ^(x-y)^ within row represent trend among treatments (0.05 < *P* ≤ 0.10).

^1^F0 Dorset ewes pregnant with twins were fed 100%, 60%, or 140% of NRC requirements from day 30 of gestation until parturition. Grandoffspring (F2) are referred to as CON-F2, RES-F2, and OVER-F2, respectively.

^2^Largest SEM across treatments for each timepoint.

^3^ *P*-value for main effect of treatment at each timepoint.

^4^Offspring were weighed weekly for the first month and every 14 d until day 252 of age. Offspring were weaned at 60 d of age.

At days 0 and 120, body morphometrics (CRL, HG, and HH) were collected, and at day 120, offspring BCS were recorded. A tendency for a main effect of sex was detected (*P *= 0.07) where ewe lambs tended to have greater BCS (3.01 ± 0.04) compared with ram lambs (2.90 ± 0.04; *P* = 0.07); however, there were no detected effects of granddam diet on BCS at days 0 or 120 (*P = *0.71). Ram HG (63.80 cm ± 0.59) and HH (51.90 cm ± 0.62) tended to be greater than ewe HG (61.00 cm ± 0.54) and HH (50.60 cm ± 0.57; *P* ≤ 0.09). A three-way interaction of treatment, sex, and time was detected for CRL (*P* = 0.03) where although at day 0, CRL was similar (*P* ≥ 0.50) across treatments and sexes, at day 120, RES-F2 rams (94.50 cm ± 1.28) tended to have greater CRL than CON-F2 ewes (89.20 cm ± 1.34) and RES-F2 ewes (85.40 cm ± 1.75; *P* ≤ 0.07). Additionally, OVER-F2 ewes (92.20 cm ± 1.28) and rams (91.90 cm ± 1.54) and CON-F2 rams (94.40 cm ± 1.89) tended to have greater CRL than RES-F2 ewes (85.40 cm ± 1.75; *P* ≤ 0.06).

Male offspring were euthanized at days 285 ± 0.93 of age, and body morphometrics, organ weights, bone density, and bone length were evaluated. Rams from restricted-fed granddams tended to be 7.4% lighter compared with CON-F2 rams (*P* = 0.07; Table 4), but OVER-F2 rams did not differ (*P* ≥ 0.42). We did not detect an effect of granddam diet (*P *≥ 0.25) on any other morphometric measurements (BCS, CRL, HG, scrotal circumference, backfat, and loin eye area). We also did not detect an effect of diet on F2 ram offspring organs or muscle weights (*P* ≥ 0.15; Table 4). Bone mineral density [CON (1.07 g/cm^2^ ± 0.02); RES (1.03 g/cm^2^ ± 0.02); OVER (1.08 g/cm^2^ ± 0.02)], femur length [CON (74.0 cm ± 1.2); RES (73.0 cm ± 0.8); OVER (71.5 cm ± 1.0)], and tibia length [CON (87.1 cm ± 1.3); RES (86.0 cm ± 0.9); OVER (85.9 cm ± 1.1)] did not differ as a result of granddam diet (*P* ≥ 0.17).

**Table 4.** Impact of poor maternal nutrition of F2 ram offspring body morphometrics and organ weights at day 285 of age

	Treatment^1^				
Item	CON-F2	RES-F2	OVER-F2	SEM^2^	*P*-Value^3^
*Body morphometrics*					
BW, kg	93.61^x^	86.69^y^	90.12^xy^	3.35	0.07
BCS	3.00^q^	3.00^q^	2.97^qq^	0.03	0.25
CRL, cm	115.00^q^	114.48^q^	111.53^qq^	1.63	0.51
HG, cm	107.63^q^	109.85^q^	108.63^qq^	1.44	0.60
Scrotal circumference, cm	33.17^q^	34.04^q^	33.06^qq^	0.67	0.57
Backfat, cm	0.35^q^	0.27^q^	0.32^qq^	0.05	0.35
LEA, cm^2^	94.83^q^	92.82^q^	102.72^qq^	9.26	0.35
*Organ weights* ^4^					
LM, g/kg BW	11.45^q^	10.81^q^	11.60^qq^	0.43	0.51
STN, g/kg BW	2.86^q^	2.80^q^	3.24^qq^	0.46	0.27
TB, g/kg BW	1.04^q^	1.07^q^	1.07^qq^	0.05	0.84
Heart, g/kg BW	3.88^q^	3.91^q^	3.85^qq^	0.30	0.35
Liver, g/kg BW	13.57^q^	15.44^q^	13.92^qq^	0.75	0.25
Pancreas, g/kg BW	0.64^q^	0.62^q^	0.71^qq^	0.08	0.61
Adrenal Gland, g/kg BW	0.02^q^	0.02^q^	0.02^qq^	0.00	0.80
Kidney, g/kg BW	1.33^q^	1.55^q^	1.90^qq^	0.46	0.15
Testes, g/kg BW	2.77^q^	2.99^q^	2.98^qq^	0.21	0.63

Means with different superscripts ^(x-y)^ within row represent trend among treatments (0.05 < *P* ≤ 0.10.

^1^F0 Dorset ewes pregnant with twins were fed 100%, 60%, or 140% of NRC requirements from day 30 of gestation until parturition. Grandoffspring (F2) are referred to as CON-F2, RES-F2, and OVER-F2, respectively. Male offspring were necropsied at day 285 of age for tissue collection.

^2^Largest SEM across treatments for each variable.

^3^ *P*-value for main effect of treatment.

^4^Organ weights are expressed as g/kg BW to account for differences in BW between treatment groups.

CRL, crown rump length; HG, heart girth; LEA, loin eye area; LM, longissimus muscle; STN, semitendinosus; TB, triceps brachii.

#### RFI trial.

All F2 offspring were subjected to a feeding trial at d 167 ± 0.33 of age to determine RFI. During the 77-d RFI trial, we did not detect an effect of granddam diet on the initial BW (*P* = 0.28; Table 5). However, F2 offspring sex influenced the initial BW, where F2 rams weighed 10.46 kg more than F2 ewes (*P* ≤ 0.0001). Final BW tended to be influenced by granddam diet (*P* = 0.09) and was influenced by offspring sex (*P* < 0.0001). Specifically, RES-F2 offspring tended to be 6.9% lighter than OVER-F2 offspring at the end of the 77-d RFI trial (*P* = 0.08). Additionally, F2 rams weighed 12.46 kg more than F2 ewes (*P* < 0.0001). Offspring MMWT tended to be greater in OVER-F2 offspring (23.6 ± 0.3) and CON-F2 offspring (23.4 ± 0.4) relative to RES-F2 offspring (22.3 ± 0.4; *P* = 0.08). Offspring ADG was similar across granddam treatment groups (*P* = 0.34); however, F2 rams tended to gain 0.03 kg/d more than F2 ewes (*P* = 0.08). Despite a tendency for greater gain in rams, average daily intake tended to be 16.7% greater in CON-F2 ewes compared with CON-F2 rams (*P* = 0.07). Finally, while CON-F2 ewes (0.31 ± 0.12) tended to be less feed efficient than CON-F2 rams (−0.45 ± 0.20) and RES-F2 rams (−0.20 ± 0.13; *P* = 0.08), and OVER-F2 ewes (0.16 ± 0.12) tended to be less feed efficient than CON-F2 rams, RES-F2 ewes were similar (−0.06 ± 0.18) in feed efficiency compared with RES-F2 rams (*P* = 0.98).

**Table 5.** Effects of poor maternal nutrition on ewe and ram offspring residual feed intake (RFI)

	Treatment^1^							*P*-Value		
	CON-F2		RES-F2		OVER-F2					
Item^3^	Ewe	Ram	Ewe	Ram	Ewe	Ram	SEM^2^	Trt	Sex	Trt*Sex
Initial BW^4^, kg	47.75	62.51	45.77	57.39	50.21	58.15	2.78	0.28	<0.0001	0.23
Final BW^5^, kg	72.24	86.55	66.10	82.42	74.29	85.24	4.34	0.09	<0.0001	0.56
MMWT^6^, kg	21.56	25.34	20.29	24.33	21.91	25.28	0.59	0.03	<0.0001	0.78
ADG, kg/d	0.32	0.31	0.26	0.33	0.31	0.35	0.04	0.34	0.08	0.35
Average Daily Intake^7^, kg/d	3.83^x^	3.19^y^	3.36^xy^	3.42^xy^	3.69^xy^	3.70^xy^	0.29	0.27	0.20	0.07
RFI coefficient^8^^,^^9^, kg/d	0.31^x^	−0.45^z^	−0.06^xyz^	−0.20^yz^	0.16^xy^	0.02^xyz^	0.26	0.49	0.01	0.08

Means with different superscripts (^x-y^) within row represent trend among treatment by sex interaction (0.05 < *P* < 0.10).

^1^Dorset ewes pregnant with twins were fed 100%, 60%, or 140% of NRC requirements from day 30 of gestation until parturition. Grandoffspring (F2) are referred to as CON-F2, RES-F2, and OVER-F2, respectively. Offspring were transitioned to a complete pelleted feed at day 153 of age. At day 168 of age, offspring were allowed ad libitum feed intake for a 77-d residual feed intake trial.

^2^Largest SEM across treatments for each variable.

^3^MMWT, metabolic mid-weight; RFI, residual feed intake.

^4^Average of two consecutive body weights at the beginning of the trial.

^5^Average of two consecutive body weights at the end of the trial.

^6^Mid-point BW^0.75^.

^7^Daily intake values averaged across the 77-d feeding trial.

^8^Coeffieicent (Average daily intake—Predicted daily intake) where predicted daily intake is obtained by the regression of average daily intake on MMWT and ADG.

^9^A negative value indicates a more efficient animal.

#### Intravenous glucose tolerance test.

Plasma glucose and insulin concentrations were measured from an IV-GTT performed on lambs at days 133 ± 0.28 of age. Granddam diet did not influence glucose or insulin average concentrations, baseline concentrations, AUC, first-phase response, or insulin:glucose ratio (*P* ≥ 0.52). However, granddam diet did influence the peak response of glucose in rams, a treatment-by-time interaction was detected (*P *= 0.02), where CON-F2 rams (297 mg/dL ± 16.5) had greater glucose peak compared with RES-F2 rams (239 mg/dL ± 11.2; *P* = 0.05). Peak insulin concentrations were not influenced by granddam diet (*P* = 0.75). Offspring sex influenced all variables of insulin response evaluated (Table 6; *P* ≤ 0.01). Specifically, average insulin concentrations across all timepoints were 35% greater in F2 rams compared with F2 ewes (*P* < 0.001). Baseline insulin concentrations were 28.9% greater in F2 rams compared with F2 ewes (*P* < 0.01). Peak insulin concentrations were 40.3% greater in F2 rams compared with F2 ewes (*P* < 0.0001). Insulin AUC were 33.2% greater in F2 rams compared with F2 ewes (*P* < 0.0001). First-phase insulin response was 39.9% greater in F2 rams compared with F2 ewes (*P* < 0.001). Finally, insulin:glucose were 35.4% greater in F2 rams compared with F2 ewes (*P* < 0.0001). Despite this sexual-dimorphic insulin response, there were no effects of offspring sex on glucose concentrations in response to a glucose challenge (*P* ≥ 0.38).

**Table 6.** Plasma glucose and insulin concentrations from the intravenous glucose tolerance test (IV-GTT) performed on F2 offspring at day 133 of age^1^

Item		F2 Ewe	F2 Ram	*P*-Value^2^
Glucose				
	Average of all time points, mg/dL	156 ± 4.62	157 ± 5.00	0.93
	Baseline^3^, mg/dL	91.8 ± 3.57	92.70 ± 3.86	0.81
	Peak concentration, mg/dL	257 ± 7.42	267 ± 8.03	0.38
	AUC^4^, AU	14,966 ± 573	14,913 ± 630	0.92
Insulin				
	Average of all time points, ng/dL	0.65 ± 0.05	1.00 ± 0.06	<0.001
	Baseline^3^, ng/dL	0.27 ± 0.03	0.38 ± 0.03	0.01
	Peak concentration, ng/dL	1.02 ± 0.12	1.71 ± 0.13	<0.001
	AUC^4^, AU	82.10 ± 7.07	130.40 ± 7.79	<0.001
	First-phase response^5^, ng/mL	2.15 ± 0.24	3.58 ± 0.26	<0.001
	Insulin, ng/mL; glucose ratio, mg/dL	0.0042 ± 0.00	0.0065 ± 0.00	<0.001

^1^Dorset ewes pregnant with twins were fed 100%, 60%, or 140% of NRC requirements from day 30 of gestation until parturition. Grandoffspring (F2) are presented by sex. Offspring were subjected to IV-GTT at day 133 of age.

^2^ *P*-Value for offspring sex for each variable.

^3^Determined from plasma samples collected at −30, −15, and 0 min relative to glucose infusion.

^4^AUC, area under the curve; AU, arbitrary units.

^5^First-phase response was calculated as the sum of 2, 5, and 10-min insulin concentrations post-glucose infusion subtracted by the mean baseline insulin concentration.

#### Circulating leptin.

Serum samples collected from F2 offspring at preweaning (days 0, 7, 14, and 56) and mature (days 210 and 252) timepoints were used to determine circulating leptin concentrations. A treatment by time interaction (*P* = 0.02) was detected for preweaning leptin concentrations where at day 56, F2 offspring from restricted- and over-fed granddams had 53.5% and 61.8% less leptin compared with F2 offspring from control-fed granddams, respectively (*P *≤ 0.02; Table 7). We did not observe an effect of granddam diet at any other preweaning timepoints, nor were any effects of F2 offspring sex detected (*P* ≥ 0.40). Despite an effect of granddam diet at day 56 of age, serum leptin at day 210 [CON (11.58 ng/dL ± 1.64); RES (12.27 ng/dL ± 1.55); OVER (12.49 ng/dL ± 1.42)] and d 252 [CON (9.00 ng/dL ± 1.64); RES (12.01 ng/dL ± 1.55); OVER (7.85 ng/dL ± 1.42)] did not differ as a result of granddam diet (*P* ≥ 0.12). An interaction of sex and time was detected for mature timepoints (*P* = 0.05) where F2 ewes at d 210 (13.56 ng/dL ± 1.21), F2 rams at d 210 (10.66 ng/dL ± 1.30), and F2 ewes at day 252 (12.91 ng/dL ± 1.21) had greater serum leptin compared with F2 rams at day 252 (6.32 ng/dL ± 1.30; *P* ≤ 0.008) and F2 rams at day 210 did not differ (10.66 ng/dL ± 1.30; *P* ≥ 0.37).

**Table 7.** Concentrations of leptin in CON, RES, and OVER lamb offspring serum at days 0, 7, 14, and 56 of age

Item	Treatment^1^				*P*-Value						
	CON-F2	RES-F2	OVER-F2	SEM^2^	Trt	Sex	Day	Trt*Sex	Trt*Day	Sex*Day	Trt*Sex*Day
Preweaning^3^											
Leptin, ng/dL					0.54	0.39	0.001	0.36	0.017	0.82	0.69
Day 0	2.50	3.03	3.68	0.69							
Day 7	2.20	1.94	1.40	0.65							
Day 14	1.62	2.07	1.63	0.69							
Day 56	4.87^a^	2.26^b^	1.86^b^	0.66							

Means with different superscripts ^(a-c)^ within row and column represent trend among treatments (0.05 < *P* < 0.10**).**

^1^Dorset ewes pregnant with twins were fed 100%, 60%, or 140% of NRC requirements from day 30 of gestation until parturition. Grandoffspring (F2) are presented by the granddam (F0) dietary treatment group. Offspring are referred to as CON-F2, RES-F2, and OVER-F2, respectively. Blood samples were collected from offspring weekly for the first month of life and every 14 d after until day 252 of age. A subset of timepoints were analyzed for each animal.

^2^Largest SEM across treatments for each variable.

^3^Days 0, 7, 14, and 56.

## Discussion

Poor maternal nutrition can have negative consequences on offspring growth and metabolism that can persist across multiple generations. Instances of impaired offspring growth and metabolism because of maternal diet have been demonstrated in rodents (Zambrano et al., 2005; Benyshek et al., 2006; Pinheiro et al., 2008) and livestock species such as sheep and pigs (Braunschweig et al., 2012; Shasa et al., 2015; Pankey et al., 2017, 2021). In livestock animals used for human consumption, altered offspring growth and metabolic dysregulation can negatively impact the efficiency of production and limit available resources to sustain the growing population. In the present study, we demonstrate the persistent impacts of maternal diet (F0) on F2 offspring growth. With similar nutritional management and environment, F1 ewes (between 16 and 19 mo of age) from over-fed dams remained smaller compared with ewes from control-fed dams before and during gestation. These findings differ from a maternal obesity model in sheep in which offspring from over-fed dams are heavier at maturity (19 to 20 mo of age) compared with offspring from control-fed dams (Long et al., 2010). Interestingly, despite differences in F1 ewe BW during gestation, F2 offspring were all of similar birth weight and remained of similar BW until after weaning. Birth weight is a highly variable indicator of offspring status and changes in body composition as a result of maternal diet can occur independent of BW at birth (Hoffman et al., 2017). Newborn F2 offspring from over-fed granddams are of similar birthweight compared with F2 offspring from control-fed granddams (Shasa et al., 2015). Despite similar birthweight, newborn F2 lambs from over-fed granddams have greater body fat percentages (Shasa et al., 2015). We previously reported that F1 offspring from restricted- and over-fed F0 dams have decreased BW into maturity (Tillquist et al., 2023). In the present study, F2 offspring were of similar BW through weaning, but after weaning, RES-F2 offspring were lighter than both CON-F2 and OVER-F2. The decrease in BW in RES-F2 offspring persisted until day 252 for all offspring, and at day 282, RES-F2 rams were 6.92 kg lighter than CON-F2 rams. These findings indicate that poor maternal diet impairs offspring growth across multiple generations. Lighter animals indicate decreased product quantity and less resources available to feed the growing population. Although RES-F2 offspring were smaller, they had similar RFI following an ad libitum feeding trial. This finding is contrary to our hypothesis that poor maternal diet would decrease offspring feed efficiency. Therefore, further investigation into potential mechanisms behind impaired growth in offspring across multiple generations is warranted.

White adipose tissue produces the peptide hormone leptin, which has an important role in appetite regulation, growth, and metabolism (Dornbush and Aeddula, 2023). An early postnatal leptin peak is imperative in the development of appetite control centers in the brain (Long et al., 2011). Over-feeding (150% of NRC) during gestation eliminates the normal leptin peak in offspring between days 6 and 9 compared with offspring from control-fed ewes (Long et al., 2011). Similar results were reported when evaluating F2 offspring. Offspring from over-fed granddams did not exhibit the normal leptin peak and had greater body fat percentages at birth compared with F2 offspring from control-fed granddams, indicating multigenerational impacts of over-feeding on offspring metabolism (Shasa et al., 2015). We previously reported that F1 offspring from over-fed F0 ewes tended to have increased preweaning leptin concentrations (Tillquist et al., 2023), and we expected that F2 offspring would yield similar findings. However, grandoffspring in this experiment from over- and restricted-fed F0 ewes had decreased leptin concentrations at 56 d of age relative to grandoffspring from control-fed granddams, and these differences do not persist into select mature timepoints. Further investigation of circulating metabolic factors is warranted as increased circulating leptin is associated with increased glucose concentrations and insulin resistance (Martin et al., 2008). We previously reported that F1 offspring from over-fed dams have greater peak glucose concentrations during an IV-GTT relative to offspring from control- and restricted- fed dams and rams from control-fed ewes have increased insulin concentrations relative to ewes from over- and restricted-fed dams at 5 min post-glucose infusion (Tillquist et al., 2023). Similarly, female offspring from restricted-fed ewes have decreased insulin response relative to control- and over-fed ewes (Vonnahme et al., 2010). In addition to differences in F1 offspring, impaired glucose homeostasis in rodent models has been reported to persist into the F2 generation (Zambrano et al., 2005; Pinheiro et al., 2008) and F3 generation (Benyshek et al., 2006) due to maternal protein restriction during gestation. Additionally, in a sheep model, F2 ewes from over-fed dams have greater instance of hyperglycemia and insulin concentrations compared with F2 ewes from control-fed dams and rams from over- or control-fed dams (Pankey et al., 2017). In the present study, we did not detect an effect of granddam diet on F2 offspring glucose tolerance. Differences are likely attributed to study design as we evaluated glucose and insulin response to a glucose challenge, and reports from Pankey et al. (2017) are circulating glucose and insulin concentrations over time during a 12-wk ad libitum feeding trial. Despite minimal impact of granddam diet on offspring glucose tolerance, we report sexual-dimorphic responses to IV-GTT in the F2 population. Specifically, F2 rams had increased insulin response to glucose bolus compared with F2 ewes. Sexual-dimorphic responses to glucose challenge have been reported in rats in F2 and F3 males (Benyshek et al., 2006). Specifically, Benyshek et al. (2006) reported increased insulin:glucose ratios in F2 and F3 males compared with female F2 and F3 offspring. The F3 generation has yet to be evaluated past the birth timepoint (Pankey et al., 2021), and this area requires further investigation to determine if transgenerational effects of maternal diet are observed in livestock models.

In summary, nutrient restriction during gestation impairs offspring growth across F1 and F2 generations. Over-feeding during gestation impairs offspring growth in the F1 generation, but F2 offspring of over-fed granddams are similar to F2 control offspring at maturity. These differences in growth are independent of differences in RFI and glucose response. Further investigation is needed to determine if the effects of poor maternal diet in a sheep model are evident in the F3 generation and if parity may influence offspring response.
