# Correction to: Effects of poor maternal diet during gestation are detected in F2 offspring

**DOI:** 10.1093/tas/txaf136

**Published:** 2025-11-11

This is a correction to: N M Tillquist, S A Reed, A S Reiter, M Y Kawaida, E C Lee, S A Zinn, K E Govoni, Effects of poor maternal diet during gestation are detected in F2 offspring, *Translational Animal Science*, Volume 8, 2024, txae055, https://doi.org/10.1093/tas/txae055

In the originally published online version of this manuscript, data in Table 4 was errored being reported to be in cm instead of inches, while LEA is off by a factor of 10. Place-keeping notations “q” and “qq” were also erroneously included. Table 4 should read:

**Table 4.** Impact of poor maternal nutrition of F2 ram offspring body morphometrics and organ weights at d 285 of age

Item^4^		Treatment^1^				
		CON-F2	RES-F2	OVER-F2	SEM^2^	*P*-Value^3^
Body Morphometrics						
BW, kg		93.61^x^	86.69^y^	90.12^xy^	3.35	0.07
BCS		3.00	3.00	2.97	0.03	0.25
CRL, cm		115.00	114.48	111.53	1.63	0.51
HG, cm		107.63	109.85	108.63	1.44	0.60
Scrotal circumference, cm		33.17	34.04	33.06	0.67	0.57
Backfat, cm		0.88	0.69	0.80	0.10	0.27
LEA, cm2		30.60	29.90	33.10	2.38	0.44
Organ Weights^5^						
LM, g/kg BW		11.45	10.81	11.60	0.43	0.51
STN, g/kg BW		2.86	2.80	3.24	0.46	0.27
TB, g/kg BW		1.04	1.07	1.07	0.05	0.84
Heart, g/kg BW		3.88	3.91	3.85	0.30	0.35
Liver, g/kg BW		13.57	15.44	13.92	0.75	0.25
Pancreas, g/kg BW		0.64	0.62	0.71	0.08	0.61
Adrenal Gland, g/kg BW		0.02	0.02	0.02	0.00	0.80
Kidney, g/kg BW		1.33	1.55	1.90	0.46	0.15
Testes, g/kg BW		2.77	2.99	2.98	0.21	0.63

Means with different superscripts ^(x-y)^ within row represent trend among treatment (0.05 < *P* ≤ 0.10).

1 F0 Dorset ewes pregnant with twins were fed 100%, 60%, or 140% of NRC requirements from d 30 of gestation until parturition. Grandoffspring (F2) are referred to as CON-F2, RES-F2, and OVER-F2, respectively. Male offspring were necropsied at d 285 of age for tissue collection.

2 Largest SEM across treatments for each variable.

3 *P*-value for main effect of treatment.

4 CRL, crown rump length; HG, heart girth; LEA, loin eye area; LM, longissimus muscle; STN, semitendinosus; TB, triceps brachii.

5 Organ weights are expressed as g/kg BW to account for differences in BW between treatment groups.

There are no changes to the findings of the paper.

The emendation has been outlined only in this correction notice to preserve the version of record.

